# Understanding Challenges of Genetic Testing on Neuromuscular Disorders from the Parental Lens

**DOI:** 10.3390/jpm13121652

**Published:** 2023-11-27

**Authors:** Farheen Hakim Zada, Ahmad Hazim Syakir Ahmad Azahari, Sau Wei Wong, Adli Ali, Noor Akmal Shareela Ismail

**Affiliations:** 1 Department of Pediatric, Faculty of Medicine, Universiti Kebangsaan Malaysia, Jalan Yaacob Latif, Cheras, Kuala Lumpur 56000, Malaysia; p114202@siswa.ukm.edu.my (F.H.Z.); p115223@siswa.ukm.edu.my (A.H.S.A.A.); swwong@ppukm.ukm.edu.my (S.W.W.); adli.ali@ppukm.ukm.edu.my (A.A.); 2 Research Centre, Hospital Tunku Ampuan Besar Tuanku Aishah Rohani, UKM Specialist Children’s Hospital, Universiti Kebangsaan Malaysia, Jalan Yaacob Latif, Cheras, Kuala Lumpur 56000, Malaysia; 3 Department of Biochemistry, Faculty of Medicine, Universiti Kebangsaan Malaysia, Jalan Yaacob Latif, Cheras, Kuala Lumpur 56000, Malaysia

**Keywords:** knowledge, awareness, perception, genetic testing, genetic counseling, neuromuscular disorder, parents

## Abstract

Neuromuscular disorders, characterized by progressive muscle degeneration and weakness, present substantial challenges to both affected individuals and their families. Genetic testing assumes a pivotal role in facilitating early diagnosis, intervention, treatment, and informed family planning for these conditions. The objective of this qualitative study is to delve into the knowledge, awareness, and perceptions surrounding genetic testing within the cohort of parents caring for individuals with neuromuscular disorders in Malaysia. A semi-structured interview approach was employed to elicit data from parents of individuals diagnosed with neuromuscular disorders, encompassing those with clinical diagnoses and those diagnosed through genetic testing. Examination of the interview responses yielded nine overarching themes, which furnish invaluable insights into the perspectives of Malaysian parents concerning genetic testing. The study discerned several challenges associated with genetic testing, notably encompassing the limited awareness among parents, the financial constraints associated with genetic testing, and the perceived significance of genetic testing in the context of neuromuscular disorders. The findings suggest that the level of knowledge and awareness pertaining to genetic testing for neuromuscular disorders among parents in Malaysia varies, with initial levels of awareness ranging from relatively low to reasonably sufficient prior to and following the birth of an affected child. However, the investigation revealed that parents tended to cultivate more favorable perceptions regarding genetic testing subsequent to their experience with genetic counseling. This underscores the potential for heightened awareness and comprehension as a consequence of the personal experience of parenting an affected child confirmed through genetic testing and genetic counseling, ultimately influencing parental awareness.

## 1. Introduction

Malaysia has witnessed significant expansion in its genetic services, offering counselling, testing, and diagnosis for genetic disorders [1,2]. Hereditary diseases have reportedly increased over the years, yet people still have poor awareness of the importance of genetic testing. Genetic testing provides valuable insights for patients, aiding in early diagnosis, treatment, and prevention of hereditary disorders [2,3,4]. The identification of hereditary elements may allow for a calculation of recurrence risks and influencing treatment decisions for specific symptoms [5]. Advances in the genetic and genomic fields have resulted in gold-standard technologies that allow thorough genetic testing for genetic disorders, which are now commercially affordable compared to before [6].

Neuromuscular disorders (NMDs) hold a significant place in Malaysia’s rare disease landscape, ranking among the top ten rare diseases in the country. The Malaysian Rare Disorders Society (MRDS) defines rare diseases as those affecting less than one in every 4000 individuals in a population [7]. Several illnesses that are considered rare globally are somewhat common among Southeast Asian populations [8]. According to the World Health Organization, it was reported that neurological ailments are ranked sixth among 28 other rare disorders, while Duchenne Muscular Dystrophy (DMD)—a form of neuromuscular disease—ranks among the top ten rare diseases in Malaysia [7].

Affecting the peripheral nervous system (PNS), a neuromuscular disease encompasses crucial components of the motor unit, including the neuromuscular junction and skeletal muscle. Any impairment to these structures can result in muscle atrophy, weakening, and sensory difficulties [4,9]. These diseases can be acquired or are heavily influenced by genetic makeup [10]. Examples of neuromuscular disorders include amyotrophic lateral sclerosis (ALS), multiple sclerosis, myasthenia gravis, myopathy, myositis, and peripheral neuropathy [10]. Currently, the most prevalent neuromuscular diseases in Malaysia are Charcot-Marie-Tooth (CMT) disease (1 in 2500 live births), followed by DMD (1 in 3500 to 6000 live male births) and spinal muscular atrophy (SMA) (1 in 20,000 live births), which manifest during infancy and adolescence [9,11,12,13,14].

NMDs display unique characteristics, yet they share common features like muscle weakness, wasting, fasciculations, cramps, or muscle pain. Different types of NMDs may exhibit overlapping symptoms, requiring involvement from various specialists and numerous medical investigations, such as extensive biochemical blood tests, muscle MRI, neurophysiological assessments, muscle/nerve biopsies, lumbar puncture, and other diagnostic tests [15,16,17]. Despite these invasive efforts, the diagnostic yield of neurogenetic testing may be low even with multiple tests carried out as mentioned above. Given the substantial costs involved in molecular tests, both in terms of resources and reagents plus the time interval to generate each test result, this delays the process of establishing an accurate diagnosis. That is why genetic testing plays a vital role in facilitating the diagnostic process [17].

A comprehensive understanding of the natural history of each neuromuscular disease is vital to ensure optimal treatment for these vulnerable patients [18]. An underlying mutation must be identified by employing genetic testing to initiate comprehensive multidisciplinary care [19]. As a result, genetic testing is becoming an essential element in the management of neuromuscular disorder patients, since it allows for optimal family planning, prevention, and access to treatment or cutting-edge clinical research [16,17].

Despite the availability of genetic services in Malaysia for over 20 years, the locals’ perception of genetic testing these days remains limited [2,3]. A published report stated that Malaysia needs to strengthen its involvement in the field of genetic testing [20]. It was reported in the literature that social stigmatization, perceived bias, and cultural taboos were described as some of the key barriers to the adoption of genetic testing in Malaysia [21]. While there have been studies on other genetic diseases in Malaysia, neuromuscular disorders have received little attention. There is a research gap, which this study attempts to address by better exploring the knowledge, awareness, and perceptions of parents of neuromuscular disorder patients regarding genetic testing, as well as suggestions to influence parental awareness and decisions related to genetic testing.

## 2. Materials and Methods

### 2.1. Research Design, Sample and Data Collection

This study employed a qualitative research design among parents of patients with neuromuscular disorders via online interviews using semi-structured questions. The participants were recruited using a convenience sampling method from three sources in total: the Pediatric Neurology Clinic of Hospital Pakar Kanak-Kanak (HPKK) UKM and two organizations, namely Spinal Muscular Atrophy Malaysia (SMAM) and the Malaysian Rare Disorders Society (MRDS). Data of patients from HPKK were accessed from the hospital’s database based on their diagnosis of neuromuscular disease and were contacted via messaging service, i.e., WhatsApp. Recruitment for SMAM and MRDS was aided by the organization’s administrative team based on the established NGO’s policy on their member’s data protection.

This study recruited any parents with children with a clinical diagnosis of neuromuscular disorders, regardless the availability of molecular diagnosis. The majority of individuals who underwent genetic testing opted for whole-exome sequencing. Hence, parents of clinically diagnosed children who have not performed genetic testing were also eligible to participate in the research. The recruitment process was initiated by filtering the participants of the study based on their eligibility criteria: that they can understand and read either English or Malay (Malaysian native language) and are Malaysian residing in Malaysia. Then, an information sheet outlining the purpose, procedures, potential risks, and benefits of the study was provided to parents with NMD children. All information regarding the study was emailed to the administrative team of MRDS and SMAM, respectively. Then, those who expressed their willingness to participate in the study were invited to be part of the research.

The semi-structured interview questions (Appendix A) used in this study were adapted from a previous study [3]. To ensure the validity and comprehensiveness of the questions in the survey, a panel of experts including medical doctors and scientific professionals were asked to review and provide feedback on the content of the questionnaire. A pilot study was conducted to establish the validity and reliability of the adapted interview guide, which involved four parents of children with rare diseases. Data from the pilot test was not included in this study. However, feedback obtained from participants of the pilot study mainly suggested simplifying the questionnaire, as some parents may have difficulty comprehending the complicated questions.

The distribution of parents recruited was two parents from HPKK, ten parents from SMAM, and five parents from MRDS. The participants were then divided into two categories based on gender, leading to the formation of separate focus groups for mothers and fathers. Participants were grouped based on their preferred interview slots. A total of five focus group discussions (FGDs) were conducted between May 2023 and June 2023, of which three were focus group discussions of mothers and two were focus group discussions of fathers. This was to ensure the responses were from a homogenous group. All participants provided informed consent, both verbally and through written consent forms, before participating in the study. The participants’ privacy is treated with utmost confidentiality, and their identity is protected by using pseudonyms in the transcripts and the final report.

The discussions were structured around three main components: knowledge, awareness, and perceptions. The FGDs facilitated open and interactive conversations among the participants, allowing them to express their opinions and share their understanding of various aspects related to genetic testing. Interviews were conducted by an independent moderator (A.H.S.A.A.) to ensure effective facilitation and deeper exploration of the topics. The moderator had no prior relationship with the parents recruited in the study.

The interviews were conducted online using video conferencing platforms such as Zoom or Microsoft Teams. The interviews were conducted in a mixture of Malay and English, depending on the participants’ preferred language.

Demographic information was collected in a separate Google Form link prior to the interview. This included parents’ age, race, religion, level of education, marital status, household income, number of affected children, age of child, performed genetic test, and association to any support group. The collection of this demographic data provided a comprehensive understanding of the participants’ backgrounds.

Recruitment ceased when the first author determined that data saturation had been reached. Data saturation was defined as the point at which no new insights or perspectives were obtained in the last few interviews.

### 2.2. Data Analysis

The sessions were recorded with the permission of each participant to ensure accurate data capture. The sessions lasted between 60 to 135 min. The audiotaped recordings of the five FGDs accumulated to a total of 562 min which were transcribed non-verbatim to 55,679 words. The transcriptions provided a written account of the participants’ perspectives and formed the basis for the subsequent thematic analysis. Following that, the transcriptions were meticulously analyzed line by line and initial codes of the transcription were formed. The codes were then refined and sorted into groups of emerging themes. Emerging themes were reviewed based on the compilation of all five FGDs to form the main themes of the study. A consensus between the authors was achieved to ensure the reliability and validity of the final themes. Quotes generated from the study were in mixed languages (Malay and English) due to the nature of the talking style of Malaysians. Hence, each quote was transcribed by the first author with the help of an online translation tool without changing the context of the sentence. This study was approved by Universiti Kebangsaan Malaysia Research Ethics Committee, reference number (UKM/PPI/111/8/JEP-2022-693) and project code (UKM.FPR.SPI 800-2/27).

## 3. Results

### 3.1. Participants Description

The focus group discussions were conducted with a total of 17 participating parents, ranging in age from 28 to 47 years old. Table 1 provides an overview of the demographic data of the participants.

### 3.2. Themes

The focus group discussions revealed 32 emerging themes related to parents’ knowledge and perceptions of genetic testing. These themes were further organized into nine main categories, as shown in Table 2. Additionally, Table 3 provides a summary of quotes from various discussion sessions, with mothers denoted as “M,” fathers as “F,” and each discussion session labeled as “FGD.” The full interview transcripts can be found in Appendix B for reference.

#### 3.2.1. Theme 1: Limited Knowledge and Awareness of Genetic Testing

All parents in this study unanimously agreed that they never knew or sought knowledge on genetic testing before they had an affected child. Some parents mentioned that their genetic knowledge and awareness were only of the widely known genetic diseases such as thalassemia or Down syndrome. Even after having an affected child, most parents complain that it is still hard for them to spread awareness to others, even to their immediate family members. Parents mentioned that the general belief seems to be that if a genetic condition does not run in the family, it is probably because the mutation of the disease is only present on the parents’ chromosomes themselves, resulting in their children inheriting genetic diseases.

#### 3.2.2. Theme 2: Significance of Genetic Testing in Family Planning and Emotional Preparedness

Most parents mentioned that they could have better mental and emotional preparation if the significance of genetic testing was known previously. This is particularly true in helping them to come to terms with their child’s diagnosis and future prognosis, as well as its implications to their family’s future. The experience of undergoing their own child’s molecular diagnosis enhances the parents’ recognition of the significance of genetic testing, especially in (i) identifying potential risks; (ii) ensuring appropriate medical care; and (iii) supporting proactive family planning. That way, it enables parents to make informed decisions about family planning and healthcare interventions.

#### 3.2.3. Theme 3: The Pivotal Role of Healthcare Professionals as Trusted Guides

Almost all parents agreed that their decision on genetic testing relies on the expertise, knowledge, and guidance provided by healthcare experts. This is because of the trust parents have towards medical professionals, believing clinicians possess the necessary skills and information for accurate diagnoses and appropriate genetic testing recommendations in guiding parents in making informed decisions. Two out of the seventeen parents worked in a healthcare facility, and both agreed that their awareness towards genetic testing and centers was largely contributed to by their medical background. In fact, one of them who had undergone genetic testing attributed the experience to her/his better awareness of genetic testing. Nevertheless, there was only a single mother who conveyed her frustration regarding certain healthcare professionals’ lack of knowledge about genetic conditions, based on her encounter with a medical doctor in a hospital’s emergency department.

#### 3.2.4. Theme 4: Perception of the Accuracy and Reliability of Genetic Testing

There is a good consensus on the reliability of genetic testing for diagnosing and providing information about hereditary disorders. Individuals perceive genetic testing as a reliable diagnostic tool that provides an accurate and trustworthy result. This perception of accuracy extends not only to local testing facilities but also to testing conducted abroad, where overseas testing is seen as particularly reliable. However, one mother expressed skepticism about the reliability of a single test and emphasized the need for additional confirmation. Similarly, a father mentioned that parents may choose to have the genetic testing done at multiple locations if they feel uncertain about the initial genetic test’s findings.

#### 3.2.5. Theme 5: The Impact of Accessibility, Cost, and Government Intervention on Genetic Testing

Almost all parents have commented on the cost and availability of genetic testing and genetic centers in Malaysia. Parents said that the expensive cost of genetic testing is one of the main causes of why parents do not test themselves. Furthermore, parents suggested various methods for government interventions, such as making pre-marital genetic testing compulsory and enhancing the infrastructure and procedures of genetic testing to increase public awareness towards genetic testing.

#### 3.2.6. Theme 6: Secular Approach to Genetic Testing Decisions

All parents unanimously expressed that religion and cultural factors have minimal influence on their decisions regarding genetic testing. They tend to rely on the expertise and guidance of medical specialists, seeking medical advice rather than allowing religious views to influence their decisions.

#### 3.2.7. Theme 7: Utilizing Digital Platforms for Genetic Testing Awareness

Some parents stated that social media is an effective platform to engage a wider audience, including those with limited exposure to genetic testing. Using social media to disseminate educational content, success stories, and information about the benefits and necessity of genetic testing can assist in overcoming barriers such as low awareness and knowledge deficiencies. Notably, one parent’s success in crowdfunding a significant amount of money for their child’s hereditary condition treatment showcased the potential of social media for raising awareness and generating support. Another parent highlighted that through superhero movies. i.e., Marvel movies, and AI technology, awareness of genetic testing was obtained.

#### 3.2.8. Theme 8: The Vital Role of Support Groups in Genetic Testing

Three out of the seventeen participants said that support groups serve as essential platforms for education, sharing of information, offering emotional support, and creating a sense of community for families facing genetic illnesses. These supporting organizations have a significant impact on increasing awareness among affected families and the wider community regarding the benefits, process, and accessibility of genetic testing. Support groups enable individuals to make informed decisions about genetic testing and obtain relevant healthcare services by providing a forum for sharing experiences, knowledge, and resources.

#### 3.2.9. Theme 9: Parental Challenges in Genetic Diseases and Testing

Parents of children with genetic diseases face significant emotional, financial, relationship, communication, and practical challenges. To make informed decisions about potential future pregnancies and their children’s well-being, some mothers consider using in vitro fertilization (IVF) to determine their genetic status. In three out of five focus group discussions, participants mentioned that having a child with a genetic condition can strain family relationships, leading to disputes and blame. Extended family members sometimes exacerbate these issues by asking difficult questions, posing communication challenges, particularly when parents’ understanding of the genetic condition is limited.

## 4. Discussion

### 4.1. Principal Findings

Given the current context and its complexities concerning genetic testing, there is a pressing need to enhance the understanding of rare diseases, which often receive limited attention from policymakers and the public. The complexity of this issue is further compounded by a lack of awareness and knowledge regarding the epidemiology and impact of rare diseases [7]. Genetic testing is a rapidly evolving field with the potential to revolutionize the healthcare system. Genetic tests can be used to diagnose diseases, predict risks, and guide treatment decisions for individuals suffering from a genetic disorder [22]. In previous quantitative research, it was revealed that a minority of respondents still held negative awareness and perception regarding genetic testing for hereditary disorders, despite efforts to provide updates on knowledge [3]. In contrast, the present qualitative study aimed to investigate the knowledge, awareness, and perception of genetic testing among parents of patients with neuromuscular disorders in Malaysia. The themes identified in this study may shed light on the challenges arising from limited knowledge, low awareness, and negative perceptions that parents may have regarding genetic testing.

There is a significant lack of knowledge and awareness about genetic testing and genetic diseases among the public, which serves as a potential barrier to proper genetic testing [23]. Interestingly, parents exhibited significantly improved knowledge after experiencing a child’s genetic disorder diagnosis and subsequent information provision. Particularly in prenatal settings, parents lack information about available genetic testing procedures and options, while the public predominantly focuses on well-known genetic conditions like thalassemia or Down syndrome, displaying limited interest in rare disorders [7]. These findings underscore the pressing necessity for expanded education, awareness campaigns, and enhanced communication methods to address knowledge gaps and the limited understanding of genetic testing and genetic illnesses [23]. Efforts should prioritize improved information accessibility, public education, and parental support in discussing genetic testing and rare diseases, ultimately fostering more informed decisions, enhanced healthcare resource access, and a deeper appreciation of genetic testing’s significance for overall health and well-being [17].

The second theme underscores the profound impact of genetic testing on family planning and parental emotional preparedness. The diagnosis of a genetic condition in a child significantly heightens parental awareness regarding the importance of genetic testing. It serves not only as a diagnostic tool for symptomatic individuals but also as a screening method for identifying carriers and asymptomatic individuals [24]. This diagnosis accentuates the significance of informed family planning decisions, as parents become aware of recurring risks in future pregnancies [17]. Genetic testing and prenatal screening play a pivotal role in early diagnosis, empowering families to make informed choices and reducing the incidence of children being born with genetic abnormalities [25]. This experience heightens awareness regarding the importance of genetic testing in identifying risk factors, ensuring appropriate medical care, and enabling proactive family planning.

Furthermore, trust and reliance on healthcare professionals, particularly doctors and clinical geneticists, emerge as a significant theme in this research. Parents heavily depend on healthcare experts when making medical decisions, especially in the context of genetic testing. The belief that these professionals possess the necessary skills and knowledge to make correct diagnoses and guide patients’ decisions instils trust in them [26]. However, it is noteworthy that many healthcare providers, including nurses and physicians, often lack a sufficient understanding of genetics relevant to their practice [22,27]. This knowledge gap extends globally, impacting healthcare professionals in various regions, including sub-Saharan Africa and the United States [20,28]. Consequently, there is a growing demand for genetic professionals, such as genetic counsellors and geneticists, in several areas, including the United States and Malaysia [28,29]. This is further justified when comparing the level of knowledge, awareness, and perceptions among the public of these countries, which are similar to what this study found. The majority of the public has a low level of knowledge and awareness towards genetic testing but a positive perception towards genetic testing, as they tend to show more concerns towards it [1].

In addition, personal health experiences, like a family history of illnesses, significantly influence individuals’ awareness of genetic testing. Healthcare practitioners play a pivotal role in raising public awareness about genetic testing and its benefits, bridging the knowledge gap between individuals with and without medical backgrounds [30]. However, physicians in practice believe that the current medical curricula do not adequately equip them with the required proficiency in medical genetics and genomics, despite acknowledging the significance of this field in medical practice [31,32]. This shows the need for continuous education and training for healthcare workers in genetics to ensure they can provide accurate information and guidance to patients seeking genetic testing and related services [22,33]. Thus, this theme highlights issues related to the low genetic knowledge among healthcare professionals, the imperative for improved genetics education, the demand for more genetic professionals, and the influence of personal health experiences in raising awareness about genetic testing, collectively emphasizing the importance of trust in healthcare professionals.

It was noted that accurate molecular diagnosis carries significant implications, including guiding disease management and treatment choices, reducing psychosocial burdens, preventing unnecessary treatments and diagnostics, identifying recurrence risks for family planning, and offering participation in clinical trials and patient registries [6,16,17]. The perception of genetic testing is overwhelmingly positive, seen as a reliable tool providing precise diagnoses for hereditary disorders, both locally and abroad. Confidence in overseas testing stems from stringent regulatory oversight and competitive pricing [33]. Despite genetic advancements, some patients with suspected neuromuscular disorders still lack genetic confirmation [17]. In such cases, a broader sequencing approach, such as panel sequencing or whole-exome and whole-genome sequencing, is advisable [34]. Multigene panel testing has successfully diagnosed individuals with conditions that were previously unidentifiable, even when initial single-gene or small panel tests yielded negative results, demonstrating its importance. However, it is essential to acknowledge that various tests may yield different results, emphasizing the need for genetic counseling to manage expectations and interpret diverse outcomes [5].

Correspondingly, this study reveals a significant theme concerning the impact of accessibility, cost, and government intervention on genetic testing. It underscores the urgent need to address barriers to genetic testing by improving accessibility, reducing costs, and implementing government initiatives. High testing expenses and limited testing facilities currently deter utilization. Financial constraints pose a common obstacle to patients seeking genetic services, significantly influencing their testing decisions [22]. Recognizing the importance of premarital and prenatal screening, the study highlights the value of early detection and informed decision-making. For instance, a study in Qatar showcased government-driven premarital screening to proactively reduce the occurrence of genetic disorders and sexually transmitted diseases, illustrating the critical role of government intervention and awareness campaigns in enhancing testing services and addressing rare disease challenges [35].

In fact, a prevailing theme of a holistic approach to genetic testing decisions among parents was one of the themes uncovered in this study. Interestingly, religion and cultural factors appear to have minimal influence on these decisions. Parents overwhelmingly rely on medical experts’ advice and prioritize it over religious beliefs, even in the context of abortion. Notably, a study highlighted that in the Muslim world, pregnancy termination is generally prohibited after the first 120 days of pregnancy. However, couples who undergo prenatal genetic testing and choose to terminate the pregnancy in the case of abnormalities or potential harm to the mother may find it ethically permissible regardless of the age of the pregnancy [36]. This religious neutrality highlights the strong emphasis on medical professionals’ knowledge and guidance in making informed choices regarding genetic testing, although it acknowledges the possibility of some religious parents who may not have participated in the study.

The impact of digital platforms, including social media, pop culture, and AI technology, on genetic testing knowledge and awareness was one of the themes explored in this study. It was found that social media emerges as a powerful tool for spreading information about genetic testing, reaching diverse audiences, particularly those unfamiliar with the topic. Rare disease patients and their families actively use social media for support and knowledge sharing [37]. Pop culture, notably superhero movies, influences public perception and understanding of genetic testing, offering opportunities for education [38]. Outstanding movies in this genre include the X-Men comic book adaptations, for example, where genetic diversity is celebrated among the mutant X-Men, while other mutants are depicted as threats to society [39]. Additionally, AI technology, particularly chatbots, are employed to deliver personalized genetic information through social media and facilitate telemedicine, improving access to healthcare knowledge [40,41]. In summary, digital platforms provide effective avenues to enhance genetic testing awareness and engagement, with AI showing promise in targeted awareness campaigns [41].

Moreover, this study uncovered that support groups are pivotal in fostering understanding, empathy, and solidarity among families grappling with genetic illnesses. They serve as vital hubs for education, information sharing, and emotional backing for families dealing with these complex disorders [42]. Through these groups, affected families glean insights into the advantages, methodologies, and accessibility of genetic testing. They are empowered to make informed choices about genetic testing and are linked to appropriate healthcare resources via the robust support networks these organizations offer. In Malaysia, key advocates for rare diseases, like the Malaysian Rare Disorders Society (MRDS), Malaysia Lysosomal Diseases Association (MLDA), and Malaysia Metabolic Society (MMS), along with other smaller societies under MRDS, actively raise awareness through local conferences, campaigns, media engagement, and social media outreach, reinforcing the vital role of support groups in enhancing awareness and knowledge among those impacted by genetic illnesses [7,42].

Similarly, parents face intricate ethical and emotional dilemmas, notably concerning reproductive choices. Raising children with genetic disorders entails substantial emotional, financial, and practical burdens, especially for mothers, often prompting consideration of in vitro fertilization (IVF) for insights into future offspring’s genetic health [36]. To aid parents in navigating these intricate decisions, comprehensive healthcare services addressing emotional, medical, and ethical guidance are essential [43]. In the context of Islamic faith, IVF is permissible for Muslim women under specific conditions outlined in Sunni Islamic guidelines, emphasizing the importance of cultural and religious considerations [36]. Nonetheless, challenges extend to conveying genetic information effectively to the public, particularly when understanding is limited [7].

The presence of a genetic condition in a child can strain relationships and give rise to emotional challenges within marriages or partnerships. Disagreements, blame, and variations in coping mechanisms may arise, further exacerbating the situation. Hence, open communication, empathy, and access to counseling and support services are essential in navigating the impact of a genetic condition on a couple’s relationship. Extended family members often provide additional support, although cultural or societal norms may sometimes hinder this [44]. Additionally, raising a child with a rare genetic condition can limit parents’ independence and socialization opportunities, potentially straining their relationship and increasing the likelihood of divorce [45]. These findings point towards the need for holistic support and understanding in families dealing with genetic disorders.

### 4.2. Limitations of the Study

This study has several limitations to note. Initially, the intended recruitment was targeted to stop when 20 participants were achieved, but the study only managed to recruit 17 participants. However, data saturation was achieved when no new emerging themes were formed during the last two FGDs, suggesting that additional recruitment might not have yielded substantially new insights. The moderator for the focus group discussions was not formally trained and conducted interviews for the first time. Additionally, the translated quotes in the results section were self-translated by the first author using an online tool, which may not be as accurate as professional translation.

Furthermore, the results of this study were obtained from two neuromuscular illnesses, namely spinal muscular atrophy and Duchenne muscular dystrophy, and may not adequately represent the entire spectrum of neuromuscular disorders. This is because the study aimed to include parents from the broader spectrum of neuromuscular diseases, but faced refusals, resulting in a limited pool of participants predominantly comprising individuals associated with SMA and DMD. Consequently, the study lacks perspectives from caretakers, which could have provided valuable insights into their experiences.

To address these limitations, future studies should aim for larger sample sizes to enhance generalizability and explore a wider range of genetic diseases. Additionally, including a more diverse set of genetic disorders and seeking input from various caretakers could provide a more comprehensive understanding of parents’ knowledge and decision-making regarding genetic testing.

## 5. Conclusions

This study concludes that parents of children with neuromuscular disorders possess sufficient knowledge and awareness of genetic testing, with a positive perception of it. The study emphasizes the need to enhance knowledge, awareness, and perception of genetic testing among Malaysians, particularly parents and individuals dealing with neuromuscular disorders. However, challenges like limited awareness, communication barriers, and high testing costs must be addressed. Policymakers and healthcare providers have a vital role in expanding genetic testing options with genetic counseling sessions, promoting early diagnosis, and improving patient outcomes. Efforts to raise public awareness, address cultural and religious concerns, and provide accessible genetic testing services can enhance the personalized management and prevention of genetic neuromuscular disorders.

## Figures and Tables

**Table 1:** Participants’ demographic data.

Components		Parents (n = 17)
Gender	Male	6 (35.3%)
	Female	11 (64.7%)
Mean age	Male	29.3 (28–46)
	Female	39.3 (30–47)
Race	Malay	15 (88.2%)
	Indian	2 (11.8%)
Religion	Islam	15 (88.2%)
	Hindu	2 (11.8%)
Education level	Primary	1 (5.9%)
	Secondary	7 (41.2%)
	Tertiary	9 (52.9%)
Marital status	Married	16 (94.1%)
	Divorced	1 (5.9%)
Household income	RM1000–RM5000	12 (70.6%)
	RM5001–RM11,000	4 (23.5%)
	RM11,001–RM50,000	1 (5.9%)
Number of affected children	1 child	15 (88.2%)
	2 children	2 (11.8%)
Type of neuromuscular disease	DMD	6 (35.3%)
	SMA	11 (64.7%)
Child’s age	Less than 1 year old	1 (5.6%)
	1–6 years old	6 (33.3%)
	7–12 years old	8 (44.4%)
	13–17 years old	3 (16.7%)
Performed genetic test	Yes	15 (88.2%)
	No	2 (11.8%)
Association to support group	Yes	15 (88.2%)
	No	2 (11.8%)

**Table 2:** The summary of emerging themes based on the focus group discussions.

Emerging Themes	Main Themes
The public’s lack of understanding and awareness of genetic testing and genetic diseases, both among medical professionals and within families.	Limited knowledge and awareness on genetic testing
Some parents are unaware of procedure and options available for genetic testing, including prenatal testing.	
There is little knowledge regarding genetic illnesses unless they are well-known, such as thalassemia or Down syndrome.	
Parents may face challenges in effectively communicating genetic information to the public, especially when their own understanding is limited.	
By enhancing awareness and communication, individuals and families can make more informed decisions, access appropriate healthcare services, and foster a better understanding of the significance of genetic testing for overall health and well-being.	
The diagnosis of a child’s genetic disease has a dramatic effect on parents, raising awareness and knowledge of the significance of genetic testing.	Significance of genetic testing in family planning and emotional preparedness
Parents undergo mental and emotional preparation, as their child’s diagnosis impacts family planning decisions and highlights the need for informed choices in subsequent pregnancies.	
Parents trust the expertise, knowledge, and guidance provided by healthcare professionals when making medical decisions.	The pivotal role of healthcare professionals as trusted guides
Healthcare professionals have a significant influence on the choices and actions of individuals, especially in the context of genetic testing and related medical interventions.	
However, some parents reported that limited knowledge regarding genetic disease exists among healthcare professionals regarding every genetic disease.	
Continuous education is essential to ensure that healthcare professionals can provide accurate information, support, and guidance to patients seeking genetic testing and related services.	
Having a medical background provides individuals with insights and knowledge that serve as an eye-opener, making them more aware of the existence and accessibility of genetic testing services and dedicated centers.	
Personal health history provides knowledge on the availability of genetic testing.	
There is a strong perception that genetic testing is accurate in providing diagnoses and information about genetic conditions.	Perception of the accuracy and reliability of genetic testing
The perception of accuracy is not limited to local testing facilities but extends to overseas testing, with the belief that testing conducted abroad is particularly accurate.	
To maintain and improve public faith in the accuracy and reliability of genetic testing, clear communication, transparency, and continuing developments in genetic testing technology are crucial.	
The high cost of genetic testing, limited facilities, and the barrier it poses to utilization are major concerns.	The impact of accessibility, cost, and government intervention on genetic testing
Financial considerations and the need for affordable options are essential factors influencing individuals’ decisions regarding genetic testing.	
Early detection through pre-marital and prenatal screening, supported by government intervention and awareness initiatives, facilitates informed decision-making, raises awareness about rare diseases, and ensures access to genetic centers and alternative testing options.	
The importance of policy measures, public health initiatives, and awareness campaigns to ensure equitable access to genetic testing services and promote informed decision-making in genetic healthcare.	
Religion and cultural considerations do not play a significant role in decisions about genetic testing.	Secular approach to genetic testing decisions
Parents typically defer to medical experts and rely on their advice, seeking guidance from the medical field rather than allowing religious beliefs to determine their decisions.	
Leveraging social media can help overcome barriers such as limited awareness and knowledge gaps by delivering educational content, success stories, and information about the benefits and importance of genetic testing.	Utilizing digital platforms for genetic testing awareness
Pop culture, as exemplified by superhero movies, plays a significant role in shaping public perception and understanding of genetic testing. The portrayal of genetic testing in popular media can create curiosity and interest among the audience, providing an opportunity to educate and inform the public about the importance and benefits of genetic testing.	
AI technology, with its ability to analyze conversations and generate personalized content on social media platforms, presents a promising avenue for targeted awareness campaigns. By utilizing AI algorithms, relevant information about genetic testing can be delivered to specific audiences, increasing engagement, and understanding.	
Support groups serve as valuable platforms for education, information sharing, and emotional support, creating a sense of community for families navigating the challenges of genetic diseases.	The vital role of support groups in genetic testing
Support groups play a significant role in increasing awareness about the benefits, process, and availability of genetic testing among affected families and the broader community.	
By offering a space for sharing experiences, knowledge, and resources, support groups empower individuals to make informed decisions regarding genetic testing and access appropriate healthcare services.	
Parents face significant emotional, financial, and practical challenges in providing care and support for their children with genetic diseases.	Parental challenges in genetic diseases and testing
Considering these challenges, some mothers contemplate reproductive options such as in vitro fertilization (IVF) to gather information about the genetic status of future pregnancies.	
This reproductive consideration reflects the desire to make informed decisions regarding the health and well-being of their potential children.	
The presence of a genetic disease in a child can lead to emotional strain, disagreements, and feelings of blame within the relationship.	
Couples may experience difficulties in navigating the complexities of the situation, including assigning responsibility for the child’s condition, or dealing with differences in coping mechanisms.	
The presence of a special child in some parents' live have also led to cases of divorce due to blaming issues.	

**Table 3:** Quotes from different FGD sessions to highlight the main themes.

Main Themes	Quote	Respondent
Limited knowledge and awareness of genetic testing	“I really don’t know about genetic testing.”	M1FGD4
	“In Malaysia, there is a slight lack of awareness during pregnancy regarding the necessary tests to be conducted. Most of the time, only blood tests such as for thalassemia are emphasized, and that is what I recently learned. Sometimes, thalassemia is the only genetic condition that receives significant attention, while other conditions are not given much consideration by people.”	M2FGD4
	“We often attribute genetic diseases to our siblings, saying that it runs in the family. But if we look back at our family history, there is no such genetic disease. So, they also act as if there is nothing to worry about. They take it lightly and don’t consider the seriousness of this disease.”	M2FGD1
Significance of genetic testing in family planning and emotional preparedness	“We want to find out if our next generation, our future children, will have the same illness as our current child.”	M1FGD2
	“It’s good to have this test… we should know early in the pregnancy… we don’t want to find out after giving birth that our child has the same illness again… so it’s better to test early, and if we have another child with the same illness, we would consider terminating the pregnancy to prevent them from suffering or having the same genetic condition again.”	F1FGD3
The pivotal role of healthcare professionals as trusted guides	“…. suggested by the doctor… offer the test for free so.… I can accept it right away. Both my wife and I want to have it done for our child because we need confirmation.”	F2FGD5
	“Doctor said, “Oh, this is the first time I’ve heard of SMA.”	M4FGD1
	“I work at a hospital. I have heard about that.”	M3FGD1
	“I underwent genetic testing when I had cancer. Because I am a breast cancer patient, and after the removal of the cancer, another cancer was detected. Due to the presence of cancer, I had to undergo another genetic test to check the DNA of the cancer cells in my blood.”	M2FGD4
Perception of the accuracy and reliability of genetic testing	“Very true… I totally agree. But that’s the problem, the diagnosis is not in Malaysia, it’s done outside the country. So, Malaysia seems outdated.”	M1FGD2
	“What the doctor explained to me about his condition, it’s exactly what happened. Until now, everything the doctor told me about the steps and the progression of the disease has been accurate.”	M3FGD4
The impact of accessibility, cost, and government intervention on genetic testing	“It took 6 months to get the results here, but when I went to Singapore, it only took 2 weeks to get the results. Even though we pay for the test both in Singapore and Malaysia, the process and cost are faster in Singapore. Not everyone can afford to spend money on genetic testing. When it comes to money, they neglect the importance of it. This is the main reason why the public is reluctant to do genetic testing. Therefore, I think the government should analyze and address this issue.”	F3FGD3
	“Genetics is not something compulsory, and maybe in terms of cost, it can be expensive. So, we don’t have much exposure to it.”	F2FGD5
Secular approach to genetic testing decisions	“They don’t know that even in Islam, it allows, according to our fatwas, the process of termination. Maybe it’s also influenced by the lack of knowledge.”	M3FGD1
	“For me, when it comes to genetic testing for any disease, I don’t see any issue because it’s about understanding our own health. It’s not about challenging nature or religion with things like this because genetic testing doesn’t discriminate against any disease or specific religious beliefs. It’s for everyone’s benefit.”	F1FGD5
Utilizing digital platforms for genetic testing awareness	“The importance of social media is actually significant in explaining about this disease so that the public can be aware of this condition.”	F4FGD3
	“I know about genetic testing from movies, like superhero movies and movies about mutants.”	M2FGD4
	“Because maybe, like me, we often discuss about children on the phone, on WhatsApp, and friends also ask. So automatically, when you open the Instagram app, eh, this thing pops up [ads generated by AI].”	M2FGD4
The vital role of support groups in genetic testing	“I obtained a lot of information about genetics through the patient association. The association for my child’s condition…”	M3FGD4
	“The awareness and dissemination about SMA are not as comprehensive as it is for CP. I also searched for information about SMA in publications but couldn’t find any. So, we must rely on patient associations, we need to communicate with the founders to create something.”	M2FGD2
Parental challenges in genetic diseases and testing	“If given the opportunity, I will undergo IVF first, no matter what effort it takes.”	M2FGD2
	“I have faced the blame that ‘this is your fault… it’s because of your family… it’s because of your genes…”	M2FGD2
	“In a family, there will always be blame and accusations, but as a family, we need to stay strong and not pay too much attention to external influences. We know that as a husband and wife, we need to be strong no matter what.”	F1FGD5

## Data Availability

The authors confirm that the data supporting the findings of this study are available within the article.

## Appendix A

Interview questions for focus group participants

**Component**	**Questions**
Knowledge	Can you please share with us your understanding/opinion about genetic testing and how it can be used to identify a specific disease? What is your understanding/opinion on how genetic testing can identify the risk of inheriting a genetic disease that is running in a family or passing down to the next generation? (Do they know their carrier status or recurrence risk) Genetic testing can be done during pregnancy to find out whether the baby will develop diseases which is known as prenatal screening. What is your opinion on this? What are the benefits of genetic testing that you have heard of? Do you think the knowledge that you have on genetic testing is adequate?
Awareness	What is your opinion on genetic testing services availability in Malaysia and where can one seek genetic testing? Genetic disorders are caused by changes in our body information and not all genetic disorders can be cured. Are you aware of this? What is your opinion regarding the public’s view and awareness on genetic testing? Do you encounter any problem when gaining knowledge on genetic testing? (Either online info or from doctors). Do you think all genetic testing can provide an exact diagnosis? Are you aware there are some commercial genetic testing kits available online and in pharmacies, have you tried it before? (Dry blood spots by Centogene or CircleDNA)
Perception	Do you think genetic testing is important? If yes or no, why? (Does it raise any ethical concern if no) In your opinion, what are the factors involved when considering opting for genetic testing? Personally, would you consider genetic testing on yourself? (Would you do it on yourself if you haven’t) There is a common misconception that genetic testing always provides clear answers, what is your opinion on the reliability of genetic test results? Do you think it tampers with nature, religion, and their beliefs? Do you think lack of education and knowledge on genetics and genetic tests are what raised ethical issues in genetic testing? Is it important to raise awareness of genetic testing in Malaysia? What are the ways to implement government laws and policies to ensure the safe and effective use of genetic testing in Malaysia?

## Appendix B

Excerpts of the interview

**Main Themes**	**Quote**	**Respondent**
Limited knowledge and awareness on genetic testing	“…I never really thought about genetic diseases. I only know about Down’s syndrome, thalassemia, that’s about it.” “We are in the medical field, but I don’t know what SMA actually is.”	M1FGD1
	“…we ourselves also do not know the procedures for how to go about it.” “…do not know can do testing during pregnancy.” “…my siblings also said genetic disease is hereditary. If we look back at family history, there are no such conditions in our lineage. So, they also seem to treat it as if it doesn’t exist. Being ignorant and do not take the disease seriously.”	M2FGD1
	“…I feel that the knowledge for awareness regarding this is very, very limited in Malaysia.”	M3FGD1
	“…we often hear about thalassemia. That’s what we are familiar with. However, we rarely hear about rare diseases, especially the more uncommon ones.”	M4FGD1
Significance of genetic testing in family planning and emotional preparedness	“…with the availability of genetic testing, I believe that in terms of cost, it means that the occurrence of births with genetic diseases can be reduced.” “Because there is a child who is affected, it means that we need to focus on them. That way, there is no need for another pregnancy.” “…Parents can also mentally prepare themselves in advance.”	M3FGD1
	“Genetic testing is important for me. At least we can be aware of this disease. Regardless of the specific disease, at least it allows us to be aware of something. Whether we choose to prevent it or continue.”	M4FGD1
	“…we want to find out if…our next generation, our future children, will have the same illness as our current child.” “If they [public] had undergone testing from the beginning, they may not have reached the same situation as we [parents with children affected with genetic diseases] are facing now. It’s challenging to handle and care for a child in this condition. It’s not that we don’t accept it, but we also want others [public] to be aware that the number of individuals with such illnesses is increasing, and the government may not have the resources to provide sufficient support. Relying on external funds can only go so far. Some people might say…, It’s enough that we have to go through this.”	M1FGD2
	“…the benefits are significant…not only for our future but also for the well-being of our children. We all desire healthy children and wish to spare them from any illnesses. When we have a child who is unwell, it takes a toll on our emotions and sometimes leads to feelings of sadness.”	M3FGD2
The pivotal role of healthcare professionals as trusted guides	“Because we can understand better what the doctor said and everything rather than online.”	F3FGD3
	“To obtain information about genetic testing, I primarily rely on doctors.” “…when the doctor mentions specific symptoms, that’s when we start searching for more information on websites.”	F4FGD3
	“Based on the doctor’s instructions, I was asked to undergo genetic testing.”	M1FGD4
	“Before this, I really didn’t understand, I didn’t know. So, from the doctor, they explained that for SMA, they said both the mother and father are carriers…” “I live in Johor, so why do I go far to receive treatment at UKM hospital? It’s because I feel confident that the doctors there are truly supportive. They provide me with all the information, and I can ask them anything that I don’t know.”	M3FGD4
	“We follow the doctor’s advice and what they tell us…… We follow the doctor’s guidance, they inform us about the positive aspects [results], and we listen to that only.”	M4FGD4
	“…the doctor said, “Oh, this is the first time I’ve heard of SMA”…”	M4FGD1
	“I work at a hospital. I have heard about that.”	M3FGD1
	“… I have a background in pathology, that’s why. When it comes to pathology, of course, it involves blood, feces, and all that. However, the information I have obtained is more related to the reasons, perhaps due to our social interactions, like my network. So having a network of doctors and people involved in the field makes me feel like there is no problem…. So, whenever I experienced allergies, my parents would take me to undergo genetic testing, and that’s how I found out about my allergies. That was the first time I learned about genetics.”	M2FGD4
	“…what the doctor explained to me about his condition, it has happened exactly as the doctor described. Until now, the steps that the doctor informed me about have proven to be true.”	M3FGD4
Perception of the accuracy and reliability of genetic testing	“…wherever we undergo genetic testing, we can indeed obtain accurate and reliable results.”	M4FGD1
	“ Very accurate. I agree completely. But that’s the problem, the diagnosis is not in Malaysia but overseas. So, it makes Malaysia seem behind in terms of medical advancements.”	M1FGD2
	“If we talk about DNA, it is accurate because it represents ourselves, our own DNA.”	M2FGD2
	“I have sent it… The one sent abroad is accurate.”	F1FGD3
	“We have done the first genetic test, but we have not yet received an answer…. I feel that perhaps one test is not sufficient.”	M2FGD4
	“ I’ve been to Singapore and also in India, the privileges given compared to our country, our country is very, very poor.”	F3FGD3
The impact of accessibility, cost, and government intervention on genetic testing	“In Malaysia, it is necessary for every pregnancy to go through genetic testing. This is done to prevent the increase in the number of children with diseases…especially rare diseases. As we focus on thalassemia only, nowadays, in Malaysia, couples are required to undergo thalassemia testing before getting married.” “…increase the number of genetic clinics center. It means that every major hospital should have its own genetic clinic.” “The reason for spreading this awareness can be initiated in local hospitals. It means that each state should have its own genetic clinic, and from there, they can spread the awareness within that state.”	M3FGD1
	“I hope that genetic centers exist in every state.” “I feel sorry for people who have to travel from far distances, like from Kelantan…they don’t have access to major hospitals so need to travel far. Small cases, where pediatrician is available, but these cases if got one specialist also okay for them to diagnose.” “Malaysia is still relatively behind. It would be great if all these services could be provided in Malaysia because when seeking such services abroad, the costs can be quite high due to the currency exchange rates. Therefore, having these facilities in Malaysia, regardless of the specific illness, would be beneficial in reducing costs.”	M1FGD2
	“…the Malaysian government is indeed unable to afford these medications because they are imported from foreign countries…their prices are in the millions…” “I hope that the authorities will implement more programs to raise awareness among the general public.” “Genetic testing is crucial because we know there is no cure available. However, public awareness about the significance of genetic testing is also needed.” “Increase awareness to the public through channels like KK or informational materials, awareness programs…so that more people can get the awareness.”	M3FGD2
	“They may be aware but choose not to undergo the testing due to the high cost involved. In that regard, the government could provide support by subsidizing the costs of genetic testing.”	F1FGD3
	“It takes 6 months to get the results but when I went to Singapore it took 2 weeks for me to get the results. Even though we pay there and in Malaysia also we are paying. So why don’t they fasten the process and cost.” “Not everyone can afford to do the genetic test. When comes to money they neglect it. This is the main reason they [public] don’t want to do genetic tests. So, I think the government should do an analysis on this matter.”	F3FGD3
	“It is important for the government to consider making genetic testing mandatory for couples before marriage, similar to HIV and thalassemia screening. By conducting genetic testing, couples can plan ahead and emotionally prepare themselves for what may happen in the future.”	F4FGD3
	“If we were to wait for the government, it would take too long, and we were too desperate to know what was going on, so we did the blood test at Prince Court. I don’t remember where they sent the blood sample, but within a month, we received the results. That’s how I found out about genetic testing from there.”	M1FGD4
	“The most reliable channel for genetic services availability is through government hospitals.” “…I was not aware of my child’s illness early on, and because of that, my care for my first child who became ill was not adequate. It is not good because if I had known earlier, I would have known how to care for him, and his health decline could have been delayed.”	M3FGD4
Secular approach to genetic testing decisions	“…they might not be aware that in Islam, based on our fatwas (religious rulings), the process of termination is allowed. It could be due to the lack of knowledge that influences their perception.”	M3FGD1
	“This is related to genetics, which falls under the field of medical science…it is not related to religious beliefs or affiliations. Since it is a medical domain, it is important to seek advice from experts in the field.”	F4FGD3
Utilizing digital platforms for genetic testing awareness	“Like W’s [name] mother…R[name]… N’s[name] mother, A[name], and others, they share their stories on social media, which is why they receive attention and highlight. If possible, mothers should actively share about their children, not to seek sympathy, but to raise awareness. As mothers, we can become advocates for our children and their conditions, creating awareness among the community. It starts with us, as mothers, taking the initiative.”	M2FGD2
	“The true wisdom behind crowdfunding for my baby and A[name] is that it has raised awareness about rare diseases. Alhamdulillah, many people have become aware of the challenges faced by individuals with rare diseases.”	F4FGD3
	“I know about genetic testing from movies, like superhero movies and movies about mutants.” “It’s probably AI, you know, that can detect things. So suddenly, the topic of genetic testing came up.”	M2FGD4
The vital role of support groups in genetic testing	“The awareness and dissemination of information about SMA are not as widespread as they are for conditions like CP. I understand that you have searched for resources on SMA but couldn’t find much. In such cases, it would be helpful to engage with relevant associations or reach out to the founders.”	M2FGD2
	“Now we have parents’ groups and all, so I also got involved in them.”	M2FGD4
	“I obtained a lot of information about genetics through the patient support group. The patient association for my child’s condition, and also the doctors, have been very helpful.”	M3FGD4
Parental challenges in genetic diseases and testing	“Complying with appointments, training, and assessments is indeed a challenging responsibility. It requires sacrificing a lot, including time and personal efforts. We need to sacrifice for our child.” “…if given the opportunity, I would pursue IVF and make every effort to try and conceive.”	M2FGD2
	“If a child is sick, after they are born, the expenses… the expenses… not just medical expenses but the cost of taking care of them becomes very high. Plus, there’s the cost of diapers, equipment… machines… It may be beyond the parents’ capabilities. So, I think it can have an impact after the child is born.”	M3FGD2
	“I have encountered situations where people blame me, saying, “This is your fault… It’s because of your family… It’s because of your lineage.”	M2FGD2
	“When I found out about this illness, my mother-in-law asked why we didn’t get a blood test before getting married. I told her that at that time, we only had an HIV test, there were no other tests like these because we didn’t know…. when she has a grandchild who is sick like this, it’s as if they blame me for not getting checked. But you know, before we got married, we only got tested for HIV.”	M1FGD4
	“…within the family, there is often blame and finger-pointing when something goes wrong. But as a family, we need to be strong, just like a husband and wife. We should not pay too much attention to external influences around us because, in the end, we know that the strength of a husband and wife is crucial no matter what happens.”	F1FGD5
	“Sometimes they ask us various things beyond our knowledge, and it can be challenging to answer because we only understand what we know.”	M4FGD1
